# Genetic and Environmental Influences on Self-Control: Assessing Self-Control with the ASEBA Self-Control Scale

**DOI:** 10.1007/s10519-018-9887-1

**Published:** 2018-02-05

**Authors:** Yayouk E. Willems, Conor V. Dolan, Catharina E. M. van Beijsterveldt, Eveline L. de Zeeuw, Dorret I. Boomsma, Meike Bartels, Catrin Finkenauer

**Affiliations:** 1 Department of Biological Psychology, Vrije Universiteit Amsterdam, van der Boechorststraat 1, 1081 BT Amsterdam, The Netherlands; 2 Amsterdam Public Health Research Institute, Vrije Universiteit Amsterdam, Amsterdam, The Netherlands; 3 Neuroscience Campus Amsterdam, Amsterdam, The Netherlands; 4 Department of Interdisciplinary Social Science, Universiteit Utrecht, Utrecht, The Netherlands

**Keywords:** Self-control, Self-report, Teacher-report, Parent-report, ASEBA, Heritability

## Abstract

This study used a theoretically-derived set of items of the Achenbach System of Empirically Based Assessment to develop the Achenbach Self-Control Scale (ASCS) for 7–16 year olds. Using a large dataset of over 20,000 children, who are enrolled in the Netherlands Twin Register, we demonstrated the psychometric properties of the ASCS for parent-, self- and teacher-report by examining internal and criterion validity, and inter-rater and test–retest reliability. We found associations between the ASCS and measures of well-being, educational achievement, and substance use. Next, we applied the classical twin design to estimate the genetic and environmental contributions to self-control. Genetic influences accounted for 64–75% of the variance in self-control based on parent- and teacher-report (age 7–12), and for 47–49% of the variance in self-control based on self-report (age 12–16), with the remaining variance accounted by non-shared environmental influences. In conclusion, we developed a validated and accessible self-control scale, and show that genetic influences explain a majority of the individual differences in self-control across youth aged 7–16 years.

**Electronic supplementary material:**

The online version of this article (10.1007/s10519-018-9887-1) contains supplementary material, which is available to authorized users.

## Introduction

Self-control—the capacity to alter unwanted impulses and behavior, in order to bring them into agreement with internal and external standards—is consistently associated with thriving mental, social, and physical well-being among children and adults (de Ridder et al. 2012; Moffitt et al. 2011; Tangney et al. 2004). A validated scale allowing for longitudinal assessments of self-control from childhood to adolescence is needed to advance investigations of its development. A self-control scale suitable for children and adolescents should take several issues into account. First, in studying children’s development, it is important that the scale is reliable across different ages. Second, we should take into account that children develop across contexts. The school context is different than the home context, with different raters providing different information (Bartels et al. 2007), and thus afford access to different behavior and insights that may be diagnostic for self-control. It is therefore important that a scale is reliable across different informants because, on the one hand, different informants afford a richer assessment of self-control, and, on the other hand, inter-rater reliability ensures robust assessment of self-control when only one rater is available. In the present study, we propose a scale that takes these issues into account.

Why is self-control important in children? Self-control entails *the strengthening of a desired action* (e.g., concentrating on an assignment, finishing homework, paying attention during class), and the capacity to *suppress an undesired impulse* (e.g., suppress temper tantrums, avoid breaking rules at home, inhibit irritable behavior in the classroom; Tangney et al. 2004). Self-control allows children to regulate their emotions, thoughts, or behavior, and underlies many skills and competences necessary to become healthy and well-adjusted adults (de Ridder et al. 2012; Finkenauer et al. 2005). For example, low self-control in early childhood is associated with less happiness, less compliance, poorer educational achievement, and with more oppositional and deviant behaviors, such as substance use in later life (Duckworth et al. 2014; Finkenauer et al. 2005; Moffitt et al. 2011).

So far, a wide variety of questionnaires have been used to assess self-control. Some researchers use self-contained questionnaires, others select specific items of existing questionnaires. For example, Moffitt et al. (2011) assessed self-control by composing a scale of items selected from different scales, such as their Dunedin Behavioral Ratings. Their assessment included items such as “emotionally labile”, “brief attention to tasks”, and “impulsive”. Likewise, Hay and Forrest (2006) and Turner and Piquero (2002) used a scale drawing items from the Child Behavior Checklist (CBCL) (Achenbach and Rescorla 2001) in combination with items of other child behavior scales such as the Rutter Behavior Scale (Hogg et al. 1997) including items such as “temper tantrums”, “has difficulty completing activities”, and “cannot wait for things”. While there is a clear overlap in items included in these aforementioned studies, these composites of items have not yet resulted in the validation of an internationally accessible and applicable self-control scale, a crucial step to improve our understanding of self-control among children in the future. In this research, we therefore investigate whether a theoretically-derived set of items, similar to the aforementioned items, of the Achenbach System of Empirically Based Assessment (ASEBA, http://www.aseba.com) can be used to assess self-control during childhood. The ASEBA is a worldwide, frequently used, multi-informant tool applied in both scientific research and clinical practice (Achenbach and Rescorla 2001; Achenbach et al. 2002). Validating a self-control scale based on such items could have vast implications. There are multiple large population based registers (e.g. NTR, Tchad, CATSS, Generation R, TRAILS) with longitudinal ASEBA data readily available (Anckarsäter et al. 2011; Lichtenstein et al. 2007; Ormel et al. 2012; Jaddoe et al. 2012). A validated ASEBA self-control scale (ASCS) allows to calculate a score for self-control in retrospect. This richness of available longitudinal data is unique, and would be difficult to become available if self-control were to be assessed from now onwards. Additionally, the ASEBA questionnaires have been translated in over 100 languages facilitating prospective cross-cultural studies. This offers novel and exciting opportunities to examine theoretical suggestions regarding the development of self-control.

The widely used ASEBA includes the CBCL, the Youth Self-Report (YSR), and Teacher’s Report Form (TRF), which were tailored for parents, children, and teachers, respectively (Achenbach and Rescorla 2001; Achenbach et al. 2002). The ASEBA questionnaires were developed to assess child maladaptive functioning, including syndrome scales such as anxiousness, depression, somatic problems and later also applied to assess dimensions of Autism and obsessive–compulsive disorder (Achenbach and Rescorla 2001; Achenbach et al. 2002; So et al. 2013; Nelson et al. 2001). They measure comparable constructs across ages with similar item content, allowing us to select items that meet the theoretical conceptualization of self-control and that overlap in item content with existing self-control scales (Moffitt et al. 2011; Tangney et al. 2004). We selected 8 items, similar in content across informants. The current study examines the psychometric properties of this 8-item scale. Depending on the informant, we call this scale the ASCS parent-report, ASCS self-report, or the ASCS teacher-report. We refer to these questionnaires collectively as the ASEBA Self-Control Scale or ASCS.

As a first step in examining the psychometric characteristics of the ASCS, we established their internal consistency and examined its dimensionality. In the literature on self-control, the dimensionality has been subject to discussion, some arguing that self-control is a unidimensional construct (Piquero et al. 2000; Tangney et al. 2004), while others suggest that it is multi-dimensional (Duckworth and Steinberg 2015; Maloney et al. 2012; Williams et al. 2007). In addition, we tested for criterion validity of the ASCS. Next, we examined associations between the ASCS and several relevant outcomes including well-being, educational achievement (i.e., school results in math, language, education level in high-school and classroom compliance, evaluated individually), and substance use (i.e., alcohol use, drunk prevalence, smoking, evaluated individually). (Duckworth et al. 2014; Finkenauer et al. 2005; Moffitt et al. 2011). We investigated the reliability of the ASCS by testing their test–retest reliability and inter-rater reliability. A number of studies investigate the stability of self-control over time. Some find evidence supporting stability (Beaver et al. 2013), while others find evidence on malleability (Burt et al. 2014; Hay and Forrest 2006; Turner and Piquero 2002). Although increases with time and age (maturation) have been found (Casey 2015), longitudinal studies have reported substantial stability of self-control (Hennecke et al. 2014). In line with these results, we expected that self-control will predict his/her own self-control in the future to a certain extent. Specifically, we expected that a child’s level of self-control, as assessed by the ASCS at age 7, predicts his/her levels of self-control at later ages. Furthermore, we expected the mother-, father-, self- and teacher-reports to be significantly correlated, indicating agreement between informants, thus addressing the ability of the ASCS to appropriately measure self-control across contexts and informants.

Next, we looked at the genetic and environmental sources of individual differences in self-control assessed with the ASCS and estimated the heritability as a function of age, informant, and sex using the classical twin design (Boomsma et al. 2002). Previous twin studies demonstrate that self-control “runs in families” (Bridgett et al. 2015). Several small-scale studies using adolescent twin data from Add Health (http://www.cpc.unc.edu/projects/addhealth) examined the genetic and environmental contributions to the variance in self-control. These studies showed heritability estimates between 44 and 64% for adolescent self-reported self-control, with the remaining variance accounted for by non-shared environmental factors, and no sex differences (Beaver et al. 2008, 2009; Boisvert et al. 2013). These results are largely consistent with more recent adolescent twin studies, such as the study by Anokhin et al. (2011), which reported a heritability estimate of 51% for self-control in 14-year-olds, and a study by Li et al. (2014), which reported a heritability estimate of 58% for self-control in 15-year-olds. In both studies the remaining variance was is accounted for by non-shared environmental factors. Studies using parent-reports consistently show stronger genetic influences, with most heritability estimates ranging between 74 and 79% (Lemery-Chalfant et al. 2008; Li et al. 2014), and one estimate of 95% (Beaver et al. 2013). Thus far, however, twin studies on self-control included relatively small samples (ranging between 372 and 825 twin pairs), few tested sex differences, and none included informantion from father- or teacher-reports. This study adds to this line of research by analyzing data from a large group of same-sex and opposite sex twin pairs, collected by the The Netherlands Twin Register (NTR), providing heritability estimates for mother-, father-, teacher- and self-report of self-control, from age 7 to 16. We also tested sex differences applying scalar and non-scalar sex limitaton models.

## Methods

### Sample and procedure

The NTR was initiated in 1987 in the Netherlands, and follows twins from childhood to adulthood (for more details see van Beijsterveldt et al. 2013). The present study includes measures of the ASEBA-CBCL/ASEBA-TRF based on mother-, father- and teacher-report of children assessed at aged 7, aged 10 and aged 12, and measures of the ASEBA-YSR based on self-reports in children aged 12, aged 14 and aged 16. Accordingly, we assessed the reliability and validity of the ASCS based on mother-, father- and teacher-report of children aged 7, aged 10 and aged 12, and measures of self-control based on self-reports in children aged 12, aged 14 and aged 16 (the scales are consistent across ASEBA measures). The current study includes data from 24,704 7-year-olds (50.3% girls), 19,589 9/10-year-olds (50.7% girls), 16,436 12-year-olds (50.9% girls), with 1704 self-reports for 12-year olds (50.8% girls), 10,020 14-year-olds (57.6% girls) and 7566 16-year-olds (59.9% girls). Participants with a disease or handicap that interfered severely with daily functioning were excluded (N = 500). For same sex twin pairs, zygosity was based on DNA polymorphisms (N = 1578) or blood markers (N = 240). For the remaining same-sex twin pairs, zygosity was determined using parent-reported items on resemblance in appearance and confusion of the twins. In approximately 93% of the cases, zygosity was correctly classified by these items (Rietveld et al. 2000). For the main analyses, we included all teacher reports, with slightly more than half of the twins sharing the same teacher (age 7, 54%; age 10, 53%; age 12, 57% of the twins were rated by the same teacher).

### Measures

#### ASEBA

The ASEBA assessment consists of standardized questionnaires, which are completed by parents (CBCL), children themselves (YSR), and/or teachers (TRF). These questionnaires are used to rate a child’s competencies and problems in the past 6 months (for parent- and self-report), or in the past 2 months (for teacher-report). The response format of the items is a 3-point scale, with response options *not true* (coded 0), *somewhat or sometimes true* (coded 1), and *very true or often true* (coded 2). The CBCL and TRF consist of 113 items and the YSR of 112. Subsets of items are summed to create syndrome scales such as social problems, anxious depressed, and somatic complaints (Achenbach and Rescorla 2001).

#### ASCS

The ASCS is intended to measure self-control as defined by person’s ability to control his or her impulses, alter his or her emotions and thoughts, and to interrupt undesired behavioral tendencies and refrain from acting on them (Muraven and Baumeister 2000). To develop the ASCS, we followed a systematic scale development procedure for item selection. In this procedure, two subject matter experts independently assessed the relevance of each item of the ASEBA to the theoretical conceptualization of self-control (Muraven and Baumeister 2000). A third reviewer independently screened all ASEBA items selecting those corresponding to items used in earlier self-control studies. To resolve disagreement, in-depth discussion followed based the theoretical literature (Muraven and Baumeister 2000; de Ridder et al. 2012) and earlier studies including separate items to construct a self-control scale (e.g., in line with items selected by Cecil et al. 2012; Moffitt et al. 2011; Hay and Forrest 2006; Turner and Piquero 2002). As a result, 8 items were selected for the ASCS (see Table 1), with 4 items of the attention problem scale (item 4, 8, 41, 78), 4 items of the aggressive behaviour scale (item 86, 87, 95), and 1 item of the rule breaking behaviour scale (item 28).

**Table 1:** ASEBA items (and corresponding number in the ASEBA instruments) included in the ASCS

No.	Item
4	Fails to finish things he/she starts
8	Can’t concentrate, can’t pay attention for long
28	Breaks rules at home, school, or elsewhere
41	Impulsive or acts without thinking
78	Inattentive or easily distracted
86	Stubborn, sullen, or irritable
87	Sudden changes in mood or feelings
95	Temper tantrums or hot temper

Numbers are all the same for the parent, child, and teacher reports. These are items of the latest version of the ASEBA instruments

We calculated the scale score given three or fewer missing item responses (Achenbach and Rescorla 2001). In the case of one to three missing item responses, we used the person-based weighted score. Cases with more than 3 items missing were excluded (2%), not expecting to influence variables of interest considering their low prevalence. Conducting our analyses in the subsample of participants without any missing values yielded similar results. Originally, the ASEBA was set up so that higher sum scores reflect higher frequency of child problems. Extending this approach to the ASCS, higher sum scores on the ASCS correspond to lower overall levels of self-control. This is in line with earlier studies on self-control (Moffitt et al. 2011).

#### Well-being

Well-being was assessed using the Cantril ladder (Cantril 1965). Parents (age 7, 9/10, 12) and children (14, 16) rated well-being on a ten-step ‘ladder’, with the bottom ‘step’ of the ladder representing the worst possible life and the top ‘step’ indicating the best possible life. Teachers rated well-being of 7, 9/10, and 12-year old children on a 5-point scale, with response options ranging from *always or almost always unhappy* (coded 1), *more often unhappy than happy* (coded 2), *equally often happy as unhappy* (coded 3), *more often happy than unhappy* (coded 4), *almost always happy* (coded 5).

#### Conners’ Parenting Rating Scale/Teacher Rating Scale—Revised

This widely used instrument assesses the severity of behavior problems of children in the past month (Conners et al. 1998a, b). The short version consists of 27 items for parent-report and 28 items for teacher-report (reported for age 7, 9/10, 12). Items are rated on a 4-point Likert scale ranging from 0 = *not true at all* (*never, rarely*), 1 = *a little bit true* (*so now and then*), 2 = *quite true* (*often, regularly*), 3 = *very much true* (*very often*), where higher scores indicate more severe symptoms. Cronbach’s alphas were in line with the Conner’s manual, reporting Cronbach’s alphas between 0.83 and 0.85 for oppositional behavior, Cronbach’s alphas between 0.78 and 0.90 for inattention, and Cronbach’s alphas between 0.78 and 0.87 for hyperactivity (Conners et al. 1998a, b).

#### Educational achievement

Educational achievement was assessed through school results in math, language, learning problems, behaviour in class and education level in high school, evaluated separately. Parents rated children’s math and language achievement (on a 5-point scale, higher scores reflecting higher grades: 1 = *fail*, 2 = *weak*, 3 = *pass*, 4 = *good*, 5 = *excellent*), and learning problems (“did your child ever have learning problems?”, on a two-point scale, 1 = *no*, 2 = *yes*). Teachers rated compliance and task orientation of the child (“in comparison to the average student in your class, how compliant is he/she?”, “in comparison to the average student in your class, how task orientated is he/she”, 7-point scale, 1 = *much less*, 2 = *less*, 3 = *a little bit less*, 4 = *average*, 5 = *little bit more*, 6 = *more*, 7 = *much more*). Adolescents (aged 14, 16) rated their level of education. The Dutch school system divides education level according to three levels: VMBO (preparing students for vocational training), HAVO (preparing students to study at universities of applied sciences) and VWO (preparing students for university), also referred to as lower level (coded as 1), middle level (coded as 2) and higher level education (coded as 3), respectively.

#### Substance use

Adolescents (aged 14, 16) were asked how often they smoked (1 = *never*, 2 = *I quit smoking*, 3 = *I smoke once a week*, 4 = *I smoke multiple times per week* 5 = *I smoke multiple times per day*), their amount of alcohol intake per day in the weekend (1 = *less than 1 glass*, 2 = *1–2 glasses*, 3 = *3–5 glasses*, 4 = *6–10 glasses*, 5 = *11–16 glasses*, 6 = *17–20 glasses*, 6 = *17–20 glasses*, 7 = *more than 20 glasses*), and whether they had ever been drunk (0 = *never*, 1 = *1–2 times*, 2 = *3–4 times*, 3 = *5–6 times*, 4 = *7–8 times*, 5 = *9–10 times*, 6 = *11–19 times*, 7 = *20–39 times*, 8 = *more frequent*).

### Strategy of analyses

In order to examine psychometric properties of the ASCS, we tested internal consistency, dimensionality, criterion validity, inter-rater reliability, test–retest reliability, and heritability estimates. We used SPSS 21 (IBM Corp. 2012) and Mplus version 7 (Muthén and Muthén 2012) and conducted the analyses separately in children aged 7, 9/10, 12, 14, and 16, and separately for mother-, father-, self- and teacher-report. To correct for the dependency of the observations due to clustering in families, a sandwich estimator was used with weighted least squares with mean variance adjusted (WLSMV) as the estimator (Rebollo et al. 2012).

We investigated internal consistency by calculating Cronbach’s alphas. The dimensionality was examined by fitting a Multimethod-Single trait confirmatory factor model (CFA) (Campbell and Fiske 1959). This allowed us to establish whether the items measure a single factor (the single “trait” self-control) while taking into account the fact that the items are taken from different subscales within the ASEBA. In this manner, we can test the dimensionality of a model with one psychometric factor and multiple residual factors. Goodness of fit was evaluated using the Root Mean Square Error of Approximation (RMSEA), the Comparative Fit Index (CFI), and the Tucker Lewis Index (TLI). We adopted the rules of thumb that the RMSEA should be between 0.05 and 0.08 or lower (adequate fit in terms of error of approximation), and the TLI and CFI should be 0.95 or larger (Hu and Bentler 1999).

We examined criterion validity by calculating cross-sectional and longitudinal correlations between ASCS and the variables mentioned above concerning adaptive behaviors (i.e., well-being, educational achievement and substance use). Additionally, we investigated inter-rater reliability by examining the correlations between the ASCS parent-, self- and teacher-report. We investigated test–retest reliability by investigating correlations between ASCS scores over time.

Next, we estimated the heritability of self-control in a classical twin design in Mplus version’, 7 (Muthén and Muthén 2012). This design is built on the premise that differences in the resemblance between monozygotic twins (sharing approximately 100% of their DNA) and dizygotic twins (sharing 50% of their segregating genes on average) can be used to parse phenotypic trait variance into environmental and genetic components (Boomsma et al. 2002). As such, this model can be applied to estimate additive genetic (A, additive effects of alleles at multiple loci), non-additive or dominance genetic (D), common environment (C, the part of the variance that is shared by members of family), and non-shared environment (E, the part of the total variance that is unique to a certain individual) effects. We used raw-data genetic structural equation modelling with maximum likelihood estimation to perform univariate model fitting analyses to estimate the contributions of A, D or C, and E.

We first fitted a saturated model to estimate the twin correlations with their 95% confidence intervals. Based on these twin correlations an ACE or an ADE model with parameters allowed to differ between boys and girls was fitted to the data. Nested submodels were compared by hierarchic χ^2^ tests. The χ^2^ statistic was computed by subtracting—2LL (log-likelihood) for the full model from that for a reduced model (v2 = − 2LL1 − (− 2LL0)). Given that the reduced model is correct, this statistic is χ^2^ distributed with degrees of freedom (df) equal to the difference in the number of parameters estimated in the two models (Δdf = df1 − df0). In addition to the χ^2^ test statistic, Akaike’s Information Criterion (AIC = v2 − 2df) was computed to compare non-nested models. A lower AIC indicates a better the fit of the model to the observed data. Quantitative sex differences were tested by constraining the A, C/D, and E parameters to be equal across sex (Neale et al. 2006). Based on the twin correlations, we see little support for qualitative sex differences, which were therefore not modelled. When sex differences appeared to be significant, a scalar-sex limitation model was tested. In this model, a difference in total variance between boys and girls is allowed, but the relative contributions of genetic and environmental influences are equal across gender (Neale et al. 2006). In order to test the significance of A, C/D factors, we fitted models without the parameter with confidence intervals including zero.

## Results

### Internal consistency

Descriptive statistics of the ASCS are presented in Table 2. Cronbach’s alpha coefficients suggested adequate internal consistency, with coefficients ranging between 0.81 and 0.83 for ASCS parent- and teacher-reports, between 0.70 and 0.73 for ASCS self-reports.

**Table 2:** Descriptives of ASCS including means, standard deviations and sample size for each rater (mother, father, teacher, self) and age (7–16)

Age	α	Informant	*M*	*SD*	Sample size				
					MZM	DZM	MZF	DZF	DOS
7	0.82	Mother	3.46	3.10	2050	2075	2286	1906	3871
7	0.81	Father	3.06	2.90	1453	1482	1671	1300	2684
7	0.81	Teacher	2.01	2.71	881	887	992	802	1631
10	0.82	Mother	3.33	3.11	1636	1572	1867	1463	3182
10	0.82	Father	2.87	2.90	1150	1083	1299	987	2161
10	0.82	Teacher	2.15	2.87	813	770	912	705	1559
12	0.82	Mother	2.95	2.93	1411	1337	1600	1274	2676
12	0.83	Father	2.64	2.83	988	938	1142	899	1859
12	0.82	Teacher	1.88	2.69	633	608	798	560	1135
12	0.73	Self	4.38	2.98	172	157	197	144	182
14	0.73	Self	4.23	2.76	739	670	1103	837	1661
16	0.70	Self	4.37	2.69	565	461	868	666	1223

### Dimensionality

The ASCS consists of 8 items, with items derived from the attention problem scale (4 out of 10), aggressive behavior scale (3 out of 18), and rule-breaking behavior scale (1 out of 17). We specified a CFA with one psychometric (trait, denoted SC) common factor representing self-control. In addition, we included one residual factor to account for the fact that the 4 items were taken from the attention problem scale (R1_att_) and a second residual factor (R2_agg_) to account for the fact that three items were taken from the aggressive behavior scale (see Fig. S1, Supplemental Material). Statistically, this model showed good model fit for parent- and teacher-reports across all ages (see Tables S2 and S3, Supplemental Material), supporting the psychometric unidimensionality of the scale. For self-reports among adolescents aged 12–16 years, a correlation was added between the residuals of item 8 and item 78 (“can’t concentrate/can’t pay attention for long”, “inattentive, easily distracted”), because these items correlated highly. Given this addition, the model fitted well (see Tables S2 and S3, Supplemental Material). The high correlation between these items suggests that children might have more difficulties making a distinction between the subtle meaning of these 2 items than adults, making children more likely to rate them more similarly (see Table S3, Supplemental Material).

### Criterion validity

Consistent with the literature (e.g., Moffitt et al. 2011), cross-sectional associations between the ASCS and several relevant outcomes, such as well-being and educational achievement, were significant in the predicted directions (see Table 3). For example, low self-control at age 7 based on mother-report was significantly correlated with mother-rated Conners’ oppositional behavior (.67), more learning problems (.28), and lower well-being (− .36). Similarly, low self-control at age 7 based on teacher-report was significantly correlated with teacher-rated Conners’ oppositional behavior (.58), and lower well-being (− .35). It also correlated negatively with compliance (− .55), and task orientation in class (− .65), two measures that were unique to teacher-reports. These results replicated in cross-sectional correlations across ages and informants (see Table 3 for details, and Table S4 for the descriptives of measures included in tests of criterion validity).

**Table 3:** Cross-sectional correlations between low self-control and validation constructs

Age	Informant	OP	IN	HYP	WB	LP	MA	LA	CO	TO
7	Mother	0.67	0.63	0.64	− 0.36	0.28	− 0.22	− 0.25		
	Father	0.61	0.60	0.57	− 0.34	0.26	− 0.21	− 0.23		
	Teacher	0.58	0.55	0.70	− 0.35				− 0.55	− 0.65
10	Mother	0.69	0.64	0.64	− 0.39	0.28	− 0.24	− 0.26		
	Father	0.64	0.63	0.60	− 0.38	0.27	− 0.21	− 0.22		
	Teacher	0.62	0.49	0.71	− 0.43				− 0.57	− 0.63
12	Mother	0.66	0.66	0.58	− 0.35	0.30	− 0.27	− 0.29		
	Father	0.65	0.66	0.56	− 0.33	0.29	− 0.25	− 0.28		
	Teacher	0.64	0.53	0.69	− 0.32				− 0.57	− 0.60

All correlations were significant at α < 0.01. Validation constructs include oppositional (OP), inattention (IN), hyperactivity (HYP), well-being (WB), learning problems (LP), school results math (MA), school results language (LA), compliance (CO) and task orientation (TO). LP, MA and LA were unique to parent reports, hence no correlations for teacher reports and these variables. CO and TO were unique to teacher reports hence no correlations for parent reports and these variables

In addition to the cross-sectional analyses, self-control at age 7 was significantly correlated with constructs to which it should theoretically be related to at age 12 (see Table S4, Supplemental Material) and age 16 (see Table 4). For example, teacher-reported low self-control at age 7 was negatively correlated with self-reported education level at age 16 (− 0.24). Mother-, father-, and teacher-reported low self-control at age 7 were positively and significantly correlated with self-reported smoking behavior at age 16, but were not significantly correlated with self-reported alcohol intake at age 16. Self-reported low self-control at age 14 was significantly correlated with both self-reported smoking and alcohol intake at age 16. See Table S6, Supplemental Material, for descriptives of measures included in criterion validity tests.

**Table 4:** Longitudinal correlations between low self-control at age 7, 12, and 14 and validation constructs at age 16, for mother-, father-, self- and teacher-reports

Age	Informant	SM	WB	AL	DR	EL
7	Mother	0.09	− 0.08	0.04^ns^	0.00^ns^	− 0.16
7	Father	0.05	− 0.09	0.01^ns^	− 0.03^ns^	− 0.15
7	Teacher	0.11	0.01^ns^	0.03^ns^	0.00^ns^	− 0.24
12	Mother	0.13	− 0.11	0.08	0.03^ns^	− 0.28
12	Father	0.12	− 0.12	0.07	0.01^ns^	− 0.28
12	Teacher	0.19	− 0.05^ns^	0.11	0.05^ns^	− 0.34
14	Self	0.21	− 0.19	0.10	0.17	− 0.09

Non-significant correlations are illustrated with ^ns^, all other correlations are significant at α < 0.01. Validation constructs include smoking (SM), well-being (WB), alcohol-use (AL), drunk prevalence (DR) and education level (EL)

### Test–Retest Reliability and Inter-rater Reliability

Inter-rater reliability of the ASCS was assessed by correlating ASCS measures over raters and time (see Table 5). Results showed significant cross-sectional correlations between the informants, with (a) significant and strong correlations between father- and mother-reports (.66–.67), (b) significant and moderate correlations between parent- and child-reports (.40–.44), (c) significant, but lower correlations between teacher- and self-reports (.29), and (d) significant and moderate correlations between teacher- and parent-reports (.32–.40).

**Table 5:** Correlations of the ASCS scales between raters (parent-, self- and teacher-report) and over time (7,10,12,14 and 16)

Age	Informant	#	1	2	3	4	5	6	7	8	9	10	11
7	Mother	1											
	Father	2	0.66										
	Teacher	3	0.36	0.32									
10	Mother	4	0.65	0.51	0.32								
	Father	5	0.51	0.60	0.30	0.67							
	Teacher	6	0.30	0.25	0.47	0.39	0.36						
12	Mother	7	0.57	0.45	0.32	0.67	0.54	0.36					
	Father	8	0.46	0.52	0.32	0.54	0.65	0.34	0.67				
	Teacher	9	0.26	0.23	0.43	0.35	0.30	0.54	0.40	0.37			
	Child	10	0.36	0.30	0.25	0.38	0.32	0.23	0.44	0.40	0.29		
14	Child	11	0.23	0.20	0.13	0.24	0.21	0.16	0.31	0.28	0.24	0.41	
16	Child	12	0.17	0.16	0.08	0.18	0.16	0.11	0.23	0.21	0.15	0.35	0.55

All correlations are significant at α < 0.01

Longitudinal correlations of the ASCS scales across informants (intervals of 3–5 years) showed similar results, with (a) significant and moderate/strong correlations between father- and mother-reports (.45–.67), (b) significant and small/moderate correlations between parent- and child-reports (.16–.38), (c) significant and small correlations between teacher- and self-reports (.08–.25), and (d) significant and small/moderate correlations between teacher- and parent-reports (.23–.35). These findings, higher correlations between mother and father but low to moderate correlation between parent and child, parallel the cross-informant correlations reported by Achenbach et al. (2002), and earlier cross-informant studies on self-control (Duckworth and Kern 2011), confirming the inter-rater reliability of the ASCS.

To examine test–retest reliability, we investigated correlations between self-control scores measured across time within raters, with time intervals of 3–5 years (see Table 5). The results showed (a) significant and strong correlations between mother-reports from age 7–12 (.57–.67) and significant and strong correlations between father-reports from age 7–12 (.52–.65), (b) significant and moderate/strong correlations between teacher-reports from age 7–12 (.43–.54) and, (c) significant and moderate/strong correlations between self-reports from age 12–16 (.35–.55). These results are consistent with longitudinal correlations of earlier studies on self-control (e.g., Turner and Piquero 2002).

### Twin data modeling

Within-twin pair correlations of each zygosity group (MZM, DZM, MZF, DZF, and DZ opposite-sex) were inspected for initial exploration of the possible contribution of genetic and environmental factors (correlations are shown in Table 6). MZ correlations were high for all informants, with the size of the correlations being relatively stable for both males and females by parent-, teacher- and self-reports. These were approximately .70–.75 for mother-report, .70–.78 for father-report, .61–.67 for teacher-report, and .40–.57 for self-report. This stability suggests that parent-, teacher- and self-report continue to report self-control in a fairly reliable way. MZ correlations were more than twice the DZ correlations at almost all ages and all informants, except for father report at ages 7, 10, and 12 and self-reports at age 12. Thus, one would expect genetic dominance or sibling interaction to be important for mother, teacher and self-reports. We observed no statistically significant zygosity effect on the variances in our data therewith suggesting the presence of D, rather than presence of a sibling interaction effect (Eaves 1976; Rietveld et al. 2003).

**Table 6:** Twin correlations (95% confidence intervals) for self-control age 7–16 and across informants (mother-, father-, teacher- and self-report)

Age	Informant	MZM	DZM	MZF	DZF	DOS
7	Mother	0.74 (0.72–0.76)	0.34 (0.30–0.38)	0.70 (0.68–0.72)	0.32 (0.28–0.36)	0.31 (0.28–0.34)
	Father	0.75 (0.72–0.77)	0.39 (0.32–0.43)	0.73 (0.71–0.76)	0.36 (0.31–0.41)	0.32 (0.29–0.36)
	Teacher	0.61 (0.57–0.66)	0.32 (0.25–0.38)	0.63 (0.59–0.67)	0.17 (0.10–0.25)	0.27 (0.23–0.32)
10	Mother	0.73 (0.71–0.76)	0.36 (0.31–0.40)	0.71 (0.69–0.73)	0.32 (0.27–0.37)	0.32 (0.29–0.35)
	Father	0.76 (0.74–0.79)	0.35 (0.30–0.41)	0.70 (0.67–0.73)	0.40 (0.32–0.45)	0.31 (0.27–0.35)
	Teacher	0.66 (0.62–0.70)	0.33 (0.26–0.39)	0.66 (0.62–0.70)	0.27 (0.18–0.35)	0.22 (0.17–0.28)
12	Mother	0.75 (0.72–0.77)	0.34 (0.29–0.39)	0.73 (0.71–0.75)	0.37 (0.32–0.42)	0.32 (0.29–0.36)
	Father	0.78 (0.76–0.81)	0.41 (0.36–0.47)	0.73 (0.70–0.75)	0.40 (0.35–0.46)	0.35 (0.31–0.39)
	Teacher	0.67 (062–0.72)	0.35 (0.27–0.43)	0.63 (0.59–0.68)	0.31 (0.21–0.41)	0.27 (0.20–0.33)
	Self	0.57 (0.47–0.68)	0.32 (0.18–0.47)	0.40 (0.27–0.52)	0.32 (0.16–0.47)	0.03 (-0.13–0.19)
14	Self	0.44 (0.37–0.50)	0.19 (0.11–0.27)	0.52 (0.47–0.56)	0.21 (0.14–0.28)	0.16 (0.11–0.22)
16	Self	0.45 (0.38–0.53)	0.23 (0.13–0.34)	0.44 (0.38–0.50)	0.15 (0.05–0.24)	0.20 (0.13–0.28)

Subsequently, we fitted a series of models that tested for relative contribution of additive genetic (A), dominant genetic (D) or common environment (C), and unique environmental (E) influences. First, we fitted ACE and ADE models separately allowing parameters to be estimated freely across sex. Based on the lowest AIC value, we selected the best fitting model, that is an ACE or an ADE model. Second, to assess sex differences we fitted a model constraining parameters to be equal across sex. Third, when sex differences appeared, a scalar sex-limitation model was tested, allowing total variance to differ between boys and girls. Fourth, if confidence intervals of the estimated parameters included zero, we refitted the model dropping that specific parameter.

The best fitting models for mother- and teacher-ratings were ADE models with scalar sex-limitation. For self-report at age 14 and 16, an ADE model without sex differences showed the best model fit. A slightly different set of candidate models emerged for father report (age 7, 10, 12) and self-report (age 12) analyses. Comparing the AIC of an ADE and ACE model, an ACE model showed better model fit. However, the confidence intervals of C included zero, suggesting an AE-scalar model to be the final best fitting model for father-report (all ages) and an AE no sex-differences model for self-report (age 12) (see Table S6, Supplemental Material, with the data presented by informant, age and model). Important to note is that we had a limited sample size at age 12 (*N* = 1704), so it is possible that we did not have enough power to detect D at this specific age (Rietveld et al. 2003).

**Fig. 1:**
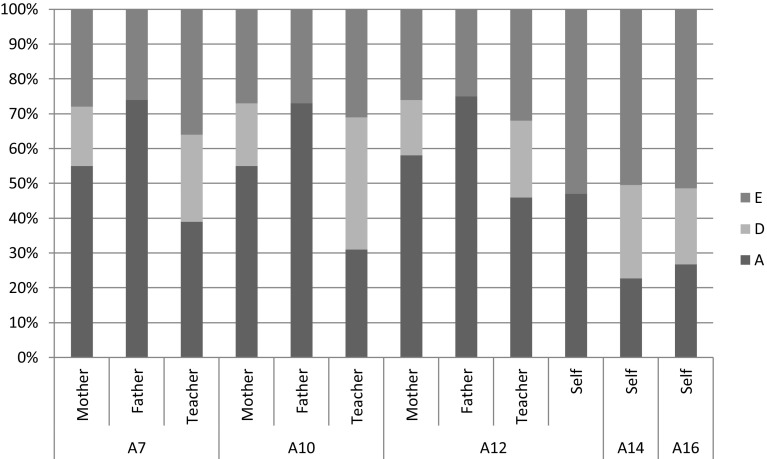
Estimates of relative contributions of genetic and environmental factors to self-control based on the best fitting model, for age 7, 10, 12, 14 and 16, for mother-, father-, teacher- and self-report, respectively

The standardized estimates of the best fitting models are presented in Fig. 1, and a full overview of estimates of the contributions of genetic and environmental factors are included in Table S7 of the Supplemental Material. For mother-, father- and teacher-report (age 7–12), genetic influences accounted for 64–75% of the variance, with unique environmental influences accounting for 25–36% of the variance in self-control. For self-report, genetic influences accounted for 47–50% of the variance in self-control age 12–16, with the remaining variance attributed to unique environmental factors. These estimates are in line with heritability estimates of earlier research (Beaver et al. 2009; Boisvert et al. 2013).

## Discussion

This study reports the construction of a self-control scale for children and adolescents, the ASCS, using the existing item pool in the widely used ASEBA questionnaires. Strengths of the study include the capitalization on widely available items to measure self-control, the use of a very large sample, the analysis of the heritability of self-control, and the examination of multiple aspects of the new scale’s psychometric functioning. The ASCS showed high internal consistency. In addition, we found high cross-sectional and longitudinal correlations between the ASCS and outcomes that were derived from the existing literature as being related to self-control (de Ridder et al. 2012; Moffitt et al. 2011), including well-being, educational achievement, and substance use. We also found that mother-, father-, self-, and teacher-reports were significantly correlated over time.

Adding to the psychometric soundness of the ASCS, we found heritability estimates paralleling earlier twin studies on self-control. A remarkable finding was that at age 12, genetic influences based on parent-reports accounted for 74–75% of the variance, while genetic influences based on self-reports accounted for only 47% of the variance. This pattern with higher heritability estimates for parent-reports than for self-reports has been reported by earlier studies on self-control and is a robust finding in the behavioral genetic literature (Anokhin et al. 2011; Beaver et al. 2009; Kan et al. 2014; Lemery-Chalfant et al. 2008). A body of empirical research attributes this finding to informant dependency; one important distinction between parent- and self-reports is that there is a single informant rating both twins (i.e., parent) versus different informants rating each twin (i.e., self-reports) (Bartels et al. 2007; Kan et al. 2014). However, the large genetic influence on self-control is in contrast to many non-genetic studies emphasizing the role of the ‘common’ environment rather than suggesting the role of genetics in the etiology of self-control, or the dynamical interaction between genes and environment. (cf. de Ridder et al. 2012; Pratt and Cullen 2000). This shows the need to bridge results from behavioral geneticists and developmental psychologists in order to investigate the underlying mechanisms of self-control development in childhood.

Gottfredson and Hirschi (1990) argue in their General Theory of Crime that self-control is formed in childhood and remains relatively stable over time (absolute levels of self-control may change over time, but an individual’s self-control relative to peers will be stable). Researchers using twin designs confirm the relative stability of self-control (cf. Beaver et al. 2008, 2013). As such, researchers in developmental psychology as well as in behavior genetics emphasize the importance of assessing self-control in youth to make inferences about adult adjustment. This is well illustrated in the recent work of Caspi et al. (2016), who assessed whether childhood risks forecast problems in adulthood. They found that children with low self-control were more likely to belong to high-cost economic burden groups as adults (e.g., using social welfare, smoking, crime, hospital stays, excessive weight). Policy makers are keen to improve well-being of adults by investing in child interventions. The returns of such an investment depends on the effectiveness of such interventions, and on the extent to which childhood outcomes predict adult adjustment. The ASCS can be used to assess self-control in youth, and thereby possibly for the prediction of adult adjustment.

In addition, the ASCS provide opportunities for secondary data-analyses. Specifically, our scale can be used to measure self-control in existing datasets, which include the ASEBA, but no questionnaires assessing self-control. Thereby, the ASCS may facilitate new research initiatives within existing research projects. For example, investigating the association between self-control and established psychopathologies or other dimensions of adult adjustment.

The results of the current study should be interpreted with some limitations in mind. While the present study has used a large population-based sample of Dutch twin youth (van Beijsterveldt et al. 2013), we recommend caution in generalizing our findings to other countries. An important next step therefore would be to replicate our findings in different populations. Vazsonyi and Belliston (2007) have investigated associations between family relationships, low self-control, and deviance across seven countries, reporting similar patterns across cultures. Furthermore, cross-cultural heritability studies for other childhood behavioral problems report on similar genetic architectures in different countries (cf. Porsch et al. 2016). Conducting comparable research would provide information on the cross-cultural validity and reliability of the ASCS. Replicating this study in twin data with a larger sample size of self-reports at age 12 is especially recommended, as research needs to clarify possible changes in environmental and genetic contributions to self-control from childhood to adolescence. Considering the international character of the ASEBA, and the wide variety of research groups including the ASEBA in their data collections, replicating this study is feasible.

Despite these limitations, the ASCS may provide insights and breakthroughs for longstanding questions and problems in the study of self-control, its links with adjustment and achievement across the lifespan, and its capacity for integration across multiple levels of analysis is especially high.

## Electronic supplementary material

Below is the link to the electronic supplementary material.

Supplementary material 1 (DOCX 174 KB)
