# Supplementary material for: Genetic and Environmental Influences on Self-Control: Assessing Self-Control with the ASEBA Self-Control Scale
Source: Behav Genet. 2018 Feb 5;48(2):135–46. doi: 10.1007/s10519-018-9887-1 (PMC5846837; doi:10.1007/s10519-018-9887-1)
Supplement: Supplementary file 1 — Supplementary material 1 (DOCX 174 KB) [file 10519_2018_9887_MOESM1_ESM.docx]

**Supplemental material (web based)**

*Figure S1.* Dimensionality ASCS, with one psychometric factor (SC, self-control) and two residual factors (R1_att_, R2_agg_).

**
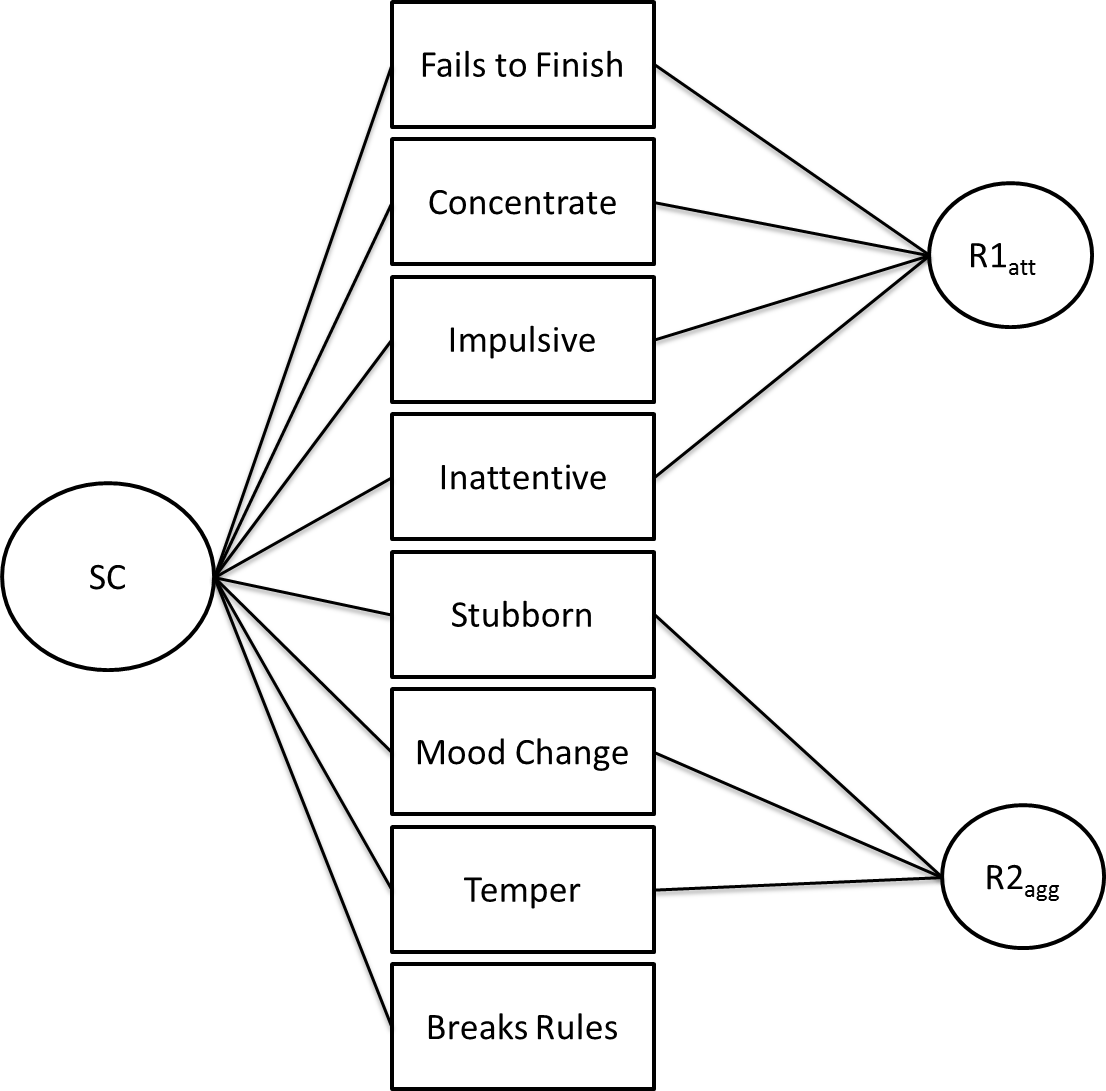
**

Table S2
*Model fit indices of ASCS for mother(MR), father (FR), teacher and self-report (SR) from age 7 till 16*

|  | A7 | | | A10 | | | A12 | | | | A14 | A16 |
| --- | --- | --- | --- | --- | --- | --- | --- | --- | --- | --- | --- | --- |
|  | MR | FR | TR | MR | FR | TR | MR | FR | TR | SR | SR | SR |
| RMSEA | 0.01 | 0.01 | 0.02 | 0.02 | 0.02 | 0.02 | 0.02 | 0.03 | 0.02 | 0.02 | 0.03 | 0.04 |
| CFI | 1.00 | 1.00 | 1.00 | 1.00 | 1.00 | 1.00 | 1.00 | 1.00 | 1.00 | 0.99 | 1.00 | 0.99 |
| TLI | 1.00 | 1.00 | 1.00 | 1.00 | 1.00 | 1.00 | 1.00 | 0.99 | 1.00 | 0.99 | 0.99 | 0.99 |

Table S3
*Standardized factor loadings of pyschometric factor Self-Control from Confirmatory Factor Analyses from age 7 to 16 for mother (MR)-,
father (FR)-, self (SR)- and teacher (TR)-report respectively, all are significant at α <.01*

|  |  | A7 | | | | | | | | | | | A10 | | | | | | | | | | | |  |
| --- | --- | --- | --- | --- | --- | --- | --- | --- | --- | --- | --- | --- | --- | --- | --- | --- | --- | --- | --- | --- | --- | --- | --- | --- | --- |
|  |  | MR | | | FR | | | | TR | | | | MR | | | | FR | | | | TR | | | |  |
| Nr. | Item Content | F | R1 | R2 | | F | R1 | R2 | | F | R1 | R2 | | F | R1 | R2 | | F | R1 | R2 | | F | R1 | R2 | |
| 4 | Fails to finish things he/she starts | .53 | .51 |  | | .55 | .50 |  | | .56 | .56 |  | | .58 | .43 |  | | .61 | .46 |  | | .56 | .55 |  | |
| 8 | Can't concentrate | .53 | .80 |  | | .52 | .83 |  | | .56 | .81 |  | | .56 | .80 |  | | .56 | .80 |  | | .56 | .81 |  | |
| 41 | Impulsive or acts without thinking | .69 | .25 |  | | .69 | .23 |  | | .78 | .25 |  | | .74 | .22 |  | | .70 | .25 |  | | .83 | .22 |  | |
| 78 | Inattentive or easily distracted | .66 | .60 |  | | .64 | .56 |  | | .64 | .64 |  | | .63 | .60 |  | | .66 | .58 |  | | .63 | .65 |  | |
| 86 | Stubborn, sullen, or irritable | .57 |  | .60 | | .56 |  | .62 | | .59 |  | .65 | | .58 |  | .62 | | .60 |  | .58 | | .65 |  | .60 | |
| 87 | Sudden changes in mood or feelings | .62 |  | .51 | | .59 |  | .55 | | .62 |  | .60 | | .62 |  | .55 | | .63 |  | .51 | | .70 |  | .52 | |
| 95 | Temper tantrums or hot temper | .60 |  | .55 | | .60 |  | .56 | | .69 |  | .57 | | .62 |  | .52 | | .62 |  | .55 | | .71 |  | .53 | |
| 28 | Breaks rules at home, school, or elsewhere | .80 |  |  | | .80 |  |  | | .89 |  |  | | .79 |  |  | | .81 |  |  | | .85 |  |  | |

|  |  | | A12 | | | | | | | | | | | | A14 | | | | | A16 | | | | | | | | | |  |  |
| --- | --- | --- | --- | --- | --- | --- | --- | --- | --- | --- | --- | --- | --- | --- | --- | --- | --- | --- | --- | --- | --- | --- | --- | --- | --- | --- | --- | --- | --- | --- | --- |
|  |  | MR | | FR | | | TR | | | SR | | | | SR | | | | | SR | | | | | | | |  |  |  |  |  |
| Nr. | Item Content | F | | R1 | R2 | F | R1 | R2 | F | R1 | R2 | F | R1 | | | R2 | F | R1 | | | R2 | | F | | R1 | | | R2 | | |  |
| 4 | Fails to finish things he/she starts | .58 | | .43 |  | .60 | .45 |  | .61 | .52 |  | .47 | .56 | | |  | .50 | .39 | | | |  | | .49 | | .27 | | |  | | |
| 8 | Can't concentrate | .57 | | .77 |  | .60 | .76 |  | .58 | .81 |  | .44 | .07 | | |  | .47 | .30 | | | |  | | .41 | | .58 | | |  | | |
| 41 | Impulsive or acts without thinking | .70 | | .28 |  | .70 | .27 |  | .83 | .22 |  | .63 | .16 | | |  | .68 | .08 | | | |  | | .65 | | .10 | | |  | | |
| 78 | Inattentive or easily distracted | .64 | | .61 |  | .66 | .57 |  | .68 | .60 |  | .61 | .35 | | |  | .66 | .24 | | | |  | | .65 | | .18 | | |  | | |
| 86 | Stubborn, sullen, or irritable | .61 | |  | .59 | .64 |  | .56 | .68 |  | .54 | .49 |  | | | .56 | .46 |  | | | | .44 | | .43 | |  | | | .45 | | |
| 87 | Sudden changes in mood or feelings | .58 | |  | .56 | .64 |  | .53 | .67 |  | .59 | .58 |  | | | .26 | .46 |  | | | | .40 | | .46 | |  | | | .34 | | |
| 95 | Temper tantrums or hot temper | .66 | |  | .47 | .64 |  | .49 | .74 |  | .51 | .57 |  | | | .31 | .54 |  | | | | .46 | | .52 | |  | | | .54 | | |
| 28 | Breaks rules at home, school, or elsewhere | .81 | |  |  | .83 |  |  | .85 |  |  | .62 |  | | |  | .62. |  | | | |  | | .58 | |  | | |  | | |

| Table S4 | |  | |  | |  | |  | |  | |  |  | |  | |  | | |
| --- | --- | --- | --- | --- | --- | --- | --- | --- | --- | --- | --- | --- | --- | --- | --- | --- | --- | --- | --- |
| Descriptives of measures included in tests of criterion validity | | | | | | | | | |  | |  |  | |  | |  | | |
| Age | Informant | | OP | | IN | | HYP | | WB | | LP | | | MA | | LA | | CO | TO |
| 7 | Mother | | 3.95 (3.37) | | 3.08 (3.80) | | 2.75 (3.26) | | 8.41 (0.96) | | 1.18 (0.38) | | | 3.69 (0.84) | | 3.60 (0.87) | |  |  |
|  | Father | | 3.94 (3.29) | | 2.88 (3.52) | | 2.88 (3.17) | | 8.36 (0.93) | | 1.15 (0.36) | | | 3.80 (0.82) | | 3.71 (0.84) | |  |  |
|  | Teacher | | 0.64 (1.54) | | 2.01 (2.91) | | 1.98 (3.30) | | 1.57 (0.73) | |  | | |  | |  | | 4.51 (1.39) | 4.79 (1.51) |
| 10 | Mother | | 3.95 (3.45) | | 3.50 (3.96) | | 2.24 (3.07) | | 8.33 (1.01) | | 1.28 (0.45) | | | 3.74 (0.95) | | 3.63 (0.93) | |  |  |
|  | Father | | 3.68 (3.33) | | 3.31 (3.77) | | 2.32 (2.95) | | 8.22 (1.01) | | 1.23 (0.42) | | | 3.76 (0.90) | | 3.69 (0.87) | |  |  |
|  | Teacher | | 0.80 (1.77) | | 2.55 (3.13) | | 1.81 (3.08) | | 1.71 (0.79) | |  | | |  | |  | | 4.60 (1.43) | 4.84 (1.58) |
| 12 | Mother | | 3.53 (3.19) | | 3.04 (3.71) | | 1.53 (2.44) | | 8.25 (1.13) | | 1.31 (0.46) | | | 3.79 (0.98) | | 3.63 (0.94) | |  |  |
|  | Father | | 3.33 (3.17) | | 2.95 (3.59) | | 1.67 (2.49) | | 8.25 (1.02) | | 1.27 (0.44) | | | 3.80 (0.95) | | 3.71 (0.91) | |  |  |
|  | Teacher | | 0.81 (1.73) | | 2.33 (2.94) | | 1.39 (2.69) | | 1.69 (0.81) | |  | | |  | |  | | 4.82 (1.44) | 5.15 (1.49) |
| Note. All correlations were significant at α <.01. Validation constructs include oppositional (OP), inattention (IN), hyperactivity (HYP), well-being (WB), learning problems (LP), school results math (MA), school results language (LA), compliance (CO) and task orientation (TO). LP, MA and LA were unique to parent reports, hence no descriptives for teacher reports and these variables. CO and TO were unique to teacher reports hence no descriptives for parent reports and these variables. | | | | | | | | | | | | | | | | | | | |

Table S5
*Longitudinal correlations between low self-control (LSC) at age 7 and
validation constructs at age 12 for mother, father, teacher report*

|  |  | | | Age 12 | | | | | | | |
| --- | --- | --- | --- | --- | --- | --- | --- | --- | --- | --- | --- |
| Age | Informant | OP | IN | | HYP | WB | LP | MA | LA | CO | TO |
| 7 | Mother | .46 | .42 | | .43 | -.23 | .26 | -.20 | -.23 |  |  |
| 7 | Father | .36 | .35 | | .35 | -.17 | .23 | -.17 | -.19 |  |  |
| 7 | Teacher | .17 | .37 | | .26 | -.11 | .31 | -.26 | -.29 | -.32 | -.41 |

*Note.* All correlations are significant at *p <.*01.Validation constructs include
oppositional (OP), inattention (IN), hyperactivity (HYP), well-being (WB),
learning problems (LP), school results math(MA), school results language
 (LA), compliance (CO) ,task orientation (TO).

Table S6
*Number of participants (N) included in longitudinal correlations*

|  |  | Age 16 | | | | | |
| --- | --- | --- | --- | --- | --- | --- | --- |
|  |  |  | SM | WB | AL | DR | EL |
|  | *M* (*SD*) |  | 1.46 (1.14) | 7.71 (1.09) | 2.61 (1.38) | 1.87 (2.25) | 1.95 (0.84) |
| Age | Informant |  |  |  |  |  |  |
| 7 | Mother |  | 4646 | 4701 | 2063 | 4737 | 4725 |
| 7 | Father |  | 3899 | 3950 | 1745 | 3970 | 3977 |
| 7 | Teacher |  | 1729 | 1802 | 1175 | 1803 | 1801 |
| 12 | Mother |  | 4217 | 4277 | 1818 | 4299 | 4300 |
| 12 | Father |  | 3338 | 3378 | 1442 | 3404 | 3396 |
| 12 | Teacher |  | 2088 | 2093 | 932 | 2117 | 2118 |
| 14 | Self |  | 3168 | 3232 | 1918 | 3244 | 3246 |

*Note.* Validation constructs include smoking (SM), well-being (WB), education level (EL),
alcohol-use (AL), drunk prevalence (DR).

| Table S7 | |  |  |  |  |  |  |  |  |
| --- | --- | --- | --- | --- | --- | --- | --- | --- | --- |
| *Model-fitting results of univariate genetic analyses of self-control (ASCS )* | | | | | | | | | |
| Age | Informant |  | Model | ep | -2LL | AIC | Comp | Δχ^2^ | *p* |
| A7 | Mother | 0 | Saturated | 25 | -58399.65 | 116849.30 | - | - | - |
|  |  | 1 | ACE sex diff | 8 | -58450.75 | 116917.50 | 0 | 51.10 (17) | <.001 |
|  |  | 2 | ADE sex diff | 8 | -58445.8 | 116907.61 | 0 | 46.15 (17) | <.001 |
|  |  | 3 | ADE no sex diff | 5 | -58527.69 | 117065.37 | 2 | 81.89 (3) | <.001 |
|  |  | **4** | **ADE scalar** | **6** | **-58454.72** | **116921.44** | **2** | **8.92 (2)** | **0.01** |
|  |  |  |  |  |  |  |  |  |  |
|  | Father | 0 | Saturated | 25 | -39959.13 | 79968.26 | - | - | - |
|  |  | 1 | ACE sex diff | 8 | -39990.04 | 79996.08 | 0 | 30.91 (17) | 0.02 |
|  |  | 2 | ADE sex diff | 8 | -39990.07 | 79996.15 | 0 | 30.94 (17) | 0.02 |
|  |  | 3 | ACE no sex diff | 5 | -40053.70 | 80117.40 | 1 | 61.30 (3) | <.001 |
|  |  | 4 | ACE scalar | 6 | -39992.40 | 79996.80 | 1 | 2.36 (2) | 0.31 |
|  |  | **5** | **AE scalar** | **5** | **-39992.40** | **79994.80** | **4** | **0.00 (1)** | **1.00** |
|  |  |  |  |  |  |  |  |  |  |
|  | Teacher | 0 | Saturated | 25 | -22419.99 | 44889.97 | - | - | - |
|  |  | 1 | ACE sex diff | 8 | -22439.8 | 44895.6 | 0 | 19.81 (17) | 0.28 |
|  |  | 2 | ADE sex diff | 8 | -22433.06 | 44882.11 | 0 | 13.07 (17) | 0.73 |
|  |  | 3 | ADE no sex diff | 5 | -22579.03 | 45168.06 | 2 | 145.97 (3) | <.001 |
|  |  | **4** | **ADE scalar** | **6** | **-22435.42** | **44882.83** | **2** | **2.36 (2)** | **0.31** |
|  |  |  |  |  |  |  |  |  |  |
| A10 | Mother | 0 | Saturated | 25 | -46708.73 | 93467.46 | - | - | - |
|  |  | 1 | ACE sex diff | 8 | -46740.68 | 93497.36 | 0 | 31.95 (17) | 0.02 |
|  |  | 2 | ADE sex diff | 8 | -46735.26 | 93486.51 | 0 | 26.53 (17) | 0.07 |
|  |  | 3 | ADE no sex diff | 5 | -46820.35 | 93650.69 | 2 | 85.09 (3) | <.001 |
|  |  | **4** | ADE scalar | **6** | **-46738.21** | **93488.43** | **2** | **2.95 (2)** | **0.23** |
|  |  |  |  |  |  |  |  |  |  |
|  | Father | 0 | Saturated | 25 | -31180.7 | 62411.39 | - | - | - |
|  |  | 1 | ACE sex diff | 8 | -31200.72 | 62417.44 | 0 | 20.02(17) | 0.27 |
|  |  | 2 | ADE sex diff | 8 | -31202.16 | 62420.31 | 0 | 21.46 (17) | 0.21 |
|  |  | 3 | ACE no sex diff | 5 | -31249.66 | 62509.32 | 1 | 48.94 (3) | <.001 |
|  |  | 4 | ACE scalar | 6 | -31209.59 | 62431.17 | 1 | 8.87 (2) | 0.02 |
|  |  | **5** | **AE scalar** | **5** | **-31209.60** | **62429.17** | **4** | **0.01 (1)** | **0.92** |
|  |  |  |  |  |  |  |  |  |  |
|  | Teacher | 0 | Saturated | 25 | -20539.2 | 41128.41 | - | - | - |
|  |  | 1 | ACE sex diff | 8 | -20566.49 | 41148.99 | 0 | 27.29 (17) | 0.05 |
|  |  | 2 | ADE sex diff | 8 | -20558.56 | 41133.11 | 0 | 19.36 (17) | 0.31 |
|  |  | 3 | ADE no sex diff | 5 | -20793.16 | 41596.33 | 2 | 234.60 (3) | <.001 |
|  |  | **4** | **ADE scalar** | **6** | **-20561.24** | **41134.48** | **2** | **2.68 (2)** | **0.26** |
|  |  |  |  |  |  |  |  |  |  |
| A12 | Mother | 0 | Saturated | 25 | -39092.18 | 78234.36 | - | - | - |
|  |  | 1 | ACE sex diff | 8 | -39128.34 | 78272.67 | 0 | 36.16 (17) | <.001 |
|  |  | 2 | ADE sex diff | 8 | -39125.55 | 78267.1 | 0 | 33.37 (17) | 0.01 |
|  |  | 3 | ADE no sex diff | 5 | -39088.56 | 78187.11 | 2 | 36.99 (3) | <.001 |
|  |  | **4** | **ADE scalar** | **6** | **-39127.69** | **78267.39** | **2** | **2.14 (2)** | **0.34** |
|  |  |  |  |  |  |  |  |  |  |
|  | Father | 0 | Saturated | 25 | -26849.6 | 53749.21 | - | - | - |
|  |  | 1 | ACE sex diff | 8 | -26863.74 | 53743.48 | 0 | 14.14 (17) | 0.66 |
|  |  | 2 | ADE sex diff | 8 | -26864.86 | 53745.72 | 0 | 15.26 (17) | 0.58 |
|  |  | 3 | ACE no sex diff | 5 | -26939.56 | 53889.11 | 1 | 75.82 (3) | <.001 |
|  |  | 4 | ACE scalar | 6 | -26869.6 | 53751.20 | 1 | 5.86 (2) | 0.05 |
|  |  | **5** | **AE scalar** | **5** | **-26870.74** | **53751.49** | **4** | **1.14 (1)** | **0.29** |
|  |  |  |  |  |  |  |  |  |  |
|  | Teacher | 0 | Saturated | 25 | -15339.62 | 30729.24 | - | - | - |
|  |  | 1 | ACE sex diff | 8 | -15369.48 | 30754.96 | 0 | 29.86 (17) | 0.03 |
|  |  | 2 | ADE sex diff | 8 | -15367.5 | 30750.99 | 0 | 27.88 (17) | 0.05 |
|  |  | 3 | ADE no sex diff | 5 | -15585.04 | 31180.08 | 2 | 217.54 (3) | <.001 |
|  |  | **4** | **ADE scalar** | **6** | **-15369.51** | **30751.01** | **2** | **2.01 (2)** | **0.37** |
|  |  |  |  |  |  |  |  |  |  |
|  | Self | 0 | Saturated | 25 | -4020.12 | 8090.23 | - | - | - |
|  |  | 1 | ACE sex diff | 7 | -4023.85 | 8061.7 | 0 | 3.73 (18) | 1.00 |
|  |  | 2 | ADE sex diff | 7 | -4026.3 | 8066.6 | 0 | 6.18 (18) | 0.98 |
|  |  | 3 | ACE no sex diff | 4 | -4030.17 | 8068.32 | 1 | 6.32 (3) | 0.10 |
|  |  | **4** | **AE no sex diff** | **3** | **-4030.20** | **8066.32** | **3** | **0.03 (1)** | **0.86** |
|  |  |  |  |  |  |  |  |  |  |
| A14 | Self | 0 | Saturated | 25 | -21880.83 | 43811.65 | - | - | - |
|  |  | 1 | ACE sex diff | 7 | -21898.04 | 43810.07 | 0 | 17.21 (18) | 0.51 |
|  |  | 2 | ADE sex diff | 7 | -21895.24 | 43804.47 | 0 | 14.41 (18) | 0.70 |
|  |  | **3** | **ADE no sex diff** | **4** | **-21899.09** | **43806.18** | **2** | **3.85 (3)** | **0.28** |
|  |  |  |  |  |  |  |  |  |  |
| A16 | Self | 0 | Saturated | 25 | -15281.66 | 30613.31 | - | - | - |
|  |  | 1 | ACE sex diff | 7 | -15309.90 | 30633.80 | 0 | 28.24 (18) | 0.06 |
|  |  | 2 | ADE sex diff | 7 | -15307.75 | 30629.49 | 0 | 26.09 | 0.09 |
|  |  | **3** | **ADE no sex diff** | **4** | **-15309.88** | **30627.75** | **2** | **2.13 (3)** | **0.55** |
| *Note:* **Bold** models were the best fitting models. | | | | | | | | | |
|  | | | | | | | | | |

Table S8
For parental, teacher and self-reports, estimates (and their 95% CI interval) of relative contributions of genetic (A, D) and environmental (E, C) factors separately for boys and girls.

| Age | Informant | Model | | **A_boys_** | **A_girls_** | **D/C_boys_** | **D/C_girls_** | **E_boys_** | **E_girls_** |
| --- | --- | --- | --- | --- | --- | --- | --- | --- | --- |
| 7 | Mother | ACE | sex diff | 0.72 ( 0.70 - 0.74) | 0.66 (0.65 - 0.68) | 0.02 (-0.01 - 0.05) | 0.03 (-0.01 - 0.06) | 0.26 (0.24 - 0.27) | 0.31 (0.29 - 0.33) |
|  |  | ADE | sex diff | 0.59 (0.21 - 0.77 ) | 0.51 (0.36 - 0.67) | 0.16 (-0.02 - 0.34) | 0.18 (0.02 - 0.34) | 0.25 ( 0.24 - 0.27) | 0.31 (0.29 - 0.33) |
|  |  | ADE | no sex diff | 0.54 (0.52 - 0.56) |  | 0.18 (0.13 - 0.23) |  | 0.28 (0.23 - 0.32) |  |
|  |  | **ADE** | **scalar** | **0.55 (0.47 - 0.63)** |  | **0.17 (0.09 - 0.25)** |  | **0.28 (0.27 - 0.29)** |  |
|  |  |  |  |  |  |  |  |  |  |
|  | Father | ACE | sex diff | 0.73 (0.70 - 0.77) | 0.72 (0.70 - 0.74) | 0.02 (-0.05 - 0.09) | 0.00 (-0.03 - 0.04) | 0.25 (0.21 - 0.29) | 0.28 (0.25 - 0.30) |
|  |  | ADE | sex diff | 0.75 (0.68 - 0.82) | 0.68 (0.54 - 0.82) | 0.01 (-0.06 - 0.07) | 0.04 (-0.09 - 0.18) | 0.25 (0.23 - 0.27) | 0.27 (0.26 - 0.29) |
|  |  | AE | no sex diff | 0.74 (0.73 - 0.75) |  |  |  | 0.26 (0.25 - 0.27) |  |
|  |  | **AE** | **scalar** | **0.74 (0.72 - 0.75)** |  |  |  | **0.26 (0.25 - 0.28)** |  |
|  |  |  |  |  |  |  |  |  |  |
|  | Teacher | ACE | sex diff | 0.59 (0.50 - 0.68) | 0.61 (0.58 - 0.64) | 0.05 (-0.03 - 0.13) | 0.01 (-0.02 - 0.03) | 0.37 (0.33 - 0.41) | 0.39 (0.37 - 0.41) |
|  |  | ADE | sex diff | 0.56 (0.37 - 0.75) | 0.29 (0.14 - 0.44) | 0.08 (-0.11 - 0.28) | 0.34 (0.19 - 0.49) | 0.36 (0.32 - 0.39) | 0.37 (0.35 - 0.39) |
|  |  | ADE | no sex diff | 0.40 (0.26 - 0.53) |  | 0.25 (0.11 - 0.39) |  | 0.36 (0.33 - 0.38) |  |
|  |  | **ADE** | **scalar** | **0.39 (0.36 - 0.43)** |  | **0.25 (0.17 - 0.32)** |  | **0.36 (0.27 - 0.46)** |  |
|  |  |  |  |  |  |  |  |  |  |
| 10 | Mother | ACE | sex diff | 0.71 ( 0.68 - 0.73) | 0.70 (0.67 - 0.72) | 0.04 ( -0.01 - 0.08) | 0.02 (-0.01 - 0.04) | 0.26 ( 0.23 - 0.28) | 0.28 (0.26 - 0.31) |
|  |  | ADE | sex diff | 0.69 (0.49 - 0.88) | 0.48 (0.37 - 0.59) | 0.06 (-0.14 - 0.26) | 0.25 (0.14 - 0.36) | 0.25 (0.24 - 0.27) | 0.28 (0.26 - 0.30) |
|  |  | ADE | no sex diff | 0.55 (0.53 - 0.56) |  | 0.19 (0.13 - 0.25) |  | 0.26 (0.21 - 0.31) |  |
|  |  | **ADE** | **scalar** | **0.55 (0.47 - 0.64)** |  | **0.18 (0.09 - 0.27)** |  | **0.27 (0.25 - 0.28)** |  |
|  |  |  |  |  |  |  |  |  |  |
|  | Father | ACE | sex diff | 0.76 (0.73 - 0.79) | 0.63 (0.53 - 0.73) | 0.00 (-0.02 - 0.02) | 0.08 (-0.02 - 0.18) | 0.24 (0.22 - 0.26) | 0.29 (0.28 0 0.31) |
|  |  | ADE | sex diff | 0.67 (0.52 - 0.82) | 0.70 (0.63 - 0.77) | 0.10 (-0.05 - 0.24) | 0.01 (-0.06 - 0.08) | 0.24 (0.21 - 0.26) | 0.29 (0.27 - 0.31) |
|  |  | AE | no sex diff | 0.74 (0.72 - 0.75) |  |  |  | 0.26 (0.25 - 0.28) |  |
|  |  | **AE** | **scalar** | **0.73 (0.72 - 0.75)** |  |  |  | **0.27 (0.25 - 0.28)** |  |
|  |  |  |  |  |  |  |  |  |  |
|  | Teacher | ACE | sex diff | 0.59 (0.56 - 0.61) | 0.64 (0.60 - 0.68) | 0.08 (0.02 - 0.15) | 0.04 (-0.01 - 0.09) | 0.33 (0.28 - 0.38) | 0.32 (0.27 - 0.37) |
|  |  | ADE | sex diff | 0.65 (0.55 - 0.75) | 0.35 (0.10 - 0.59) | 0.03 (-0.23 - 0.29) | 0.35 (0.07 - 0.63) | 0.32 (0.16 - 0.48) | 0.31 - 0.27 - 0.34) |
|  |  | ADE | no sex diff | 0.30 (0.23 - 0.36) |  | 0.40 (0.36 - 0.43) |  | 0.31 (0.22 - 0.40) |  |
|  |  | **ADE** | **scalar** | **0.31 (0.16 - 0.46)** |  | **0.38 (0.23 - 0.53)** |  | **0.31 (0.29 - 0.53)** |  |
|  |  |  |  |  |  |  |  |  |  |
|  |  |  |  |  |  |  |  |  |  |
| 12 | Mother | ACE | sex diff | 0.72 (0.69 - 0.76) | 0.69 (0.62 - 0.76) | 0.02 (-0.0 - 0.04) | 0.05 (-0.01 - 0.11) | 0.26 (0.24 - 0.28) | 0.26 (0.24 - 0.28) |
|  |  | ADE | sex diff | 0.57 (0.40 - 0.74) | 0.74 (0.66 - 0.81) | 0.17 (0.00 - 0.34) | 0.01 (-0.06 0.08) | 0.26 (0.24 - 0.28) | 0.26 (0.24 - 0.27) |
|  |  | ADE | no sex diff | 0.65 (056 - 0.74) |  | 0.09 (0.00 - 0.19) |  | 0.26 (0.24 - 0.27) |  |
|  |  | **ADE** | **scalar** | **0.58 (0.56 - 0.61)** |  | **0.16 (0.09 - 0.23)** |  | **0.26 (0.21 - 0.23)** |  |
|  |  |  |  |  |  |  |  |  |  |
|  | Father | ACE | sex diff | 0.74 (0.64 - 0.84) | 0.68 (0.57 - 0.79) | 0.04 (-0.06 - 0.14) | 0.04 (-0.07 - 0.16) | 0.22 (0.20 - 0.24) | 0.28 (0.26 - 0.29) |
|  |  | ADE | sex diff | 0.78 (0.76 - 0.80) | 0.73 (0.71 - 0.74) | 0.00 (0.00 - 0.00) | 0.00 (0.00 - 0.00) | 0.22 (0.20 - 0.24) | 0.27 (0.26 -0.29) |
|  |  | AE | no sex diff | 0.76 (0.75 - 0.77) |  |  |  | 0.24 (0.23 - 0.25) |  |
|  |  | **AE** | **scalar** | **0.75 (0.74 - 0.77)** |  |  |  | **0.25 (0.23 - 0.26)** |  |
|  |  |  |  |  |  |  |  |  |  |
|  | Teacher | ACE | sex diff | 0.61 (0.49 - 0.73) | 0.64 (0.58 - 0.69) | 0.07 (-0.03 - 0.18) | 0.02 (-0.03 - 0.07) | 0.32 (0.28 - 0.36) | 0.34 (0.32 - 0.36) |
|  |  | ADE | sex diff | 0.69 (0.65 - 0.73) | 0.39 (0.09 - 0.70) | 0.00 (-0.02 - 0.02) | 0.27 (-0.03 - 0.58) | 0.31 (0.27 - 0.35) | 0.34 (0.31 - 0.36) |
|  |  | ADE | no sex diff | 0.40 (0.36 - 0.45 ) |  | 0.29 (0.21 - 0.36) |  | 0.29 (0.21 - 0.42) |  |
|  |  | **ADE** | **Scalar** | **0.46 (0.28 - 0.63)** |  | **0.22 (0.04 - 0.40)** |  | **0.32 (0.30 - 0.35)** |  |
|  |  |  |  |  |  |  |  |  |  |
|  | Self | ACE | sex diff | 0.47 (0.41 - 0.53) | 0.22 (0.03 - 0.41) | 0.09 (-0.11 - 0.29 ) | 0.18 (0.01 - 0.35) | 0.44 (0.23 - 0.65) | 0.60 (0.26 - 0.94) |
|  |  | ADE | sex diff | 0.39 (0.23 - 0.54) | 0.26 (0.04 - 0.49) | 0.18 (-0.18 - 0.54) | 0.16 (-0.18 - 0.49) | 0.43 ( -0.05 - 0.91) | 0.58 (0.03 - 1.00) |
|  |  | **AE** | **no sex diff** | **0.47 (0.43 - 0.51)** |  |  |  | **0.53 (0.49 - 0.57)** |  |
|  |  |  |  |  |  |  |  |  |  |
| 14 | Self | ACE | sex diff | 0.37 (0.26 - 0.48) | 0.49 (0.44 - 0.55) | 0.05 (-0.03 - 0.14) | 0.02 (-0.03 - 0.07) | 0.58 (0.52 - 0.64) | 0.49 (0.47 - 0.51) |
|  |  | ADE | sex diff | 0.38 (0.18 - 0.58) | 0.32 (0.06 - 0.58) | 0.05 (0.15 - 0.25) | 0.21 (-0.06 - 0.47) | 0.57 (0.55 - 0.60) | 0.47 (0.43 - 0.52) |
|  |  | **ADE** | **no sex diff** | **0.23 (0.14 - 0.31)** |  | **0.27 (0.19 - 0.35)** |  | **0.51 (0.35 - 0.66)** |  |
|  |  |  |  |  |  |  |  |  |  |
| 16 | Self | ACE | sex diff | 0.45 (0.35 - 0.55) | 0.45 (0.37 - 0.53) | 0.04 (-0.23 - 0.30) | 0.01 (-0.15 - 0.17) | 0.51 (0.34 - 0.69) | 0.54 (0.45 - 0.63) |
|  |  | ADE | sex diff | 0.40 (0.04 - 0.76) | 0.20 (0.00 - 0.41) | 0.10 (-0.26 - 0.46) | 0.28 (0.05 - 0.50) | 0.50 (0.47 - 0.53) | 0.52 (0.47 - 0.58) |
|  |  | **ADE** | **no sex diff** | **0.27 (0.17 - 0.36)** |  | **0.22 (0.09 - 0.35)** |  | **0.52 (0.09 - 0.35)** |  |
